# An annotated image dataset for small apple fruitlet detection in complex orchard environments

**DOI:** 10.3389/fpls.2025.1664972

**Published:** 2026-01-05

**Authors:** Dandan Wang, Bo Wang

**Affiliations:** 1 College of Communication and Information Engineering, Xi’an University of Science and Technology, Xi’an, Shaanxi, China; 2 Xi’an Key Laboratory of Network Convergence Communication, Xi’an, Shaanxi, China; 3 School of Automation Science and Engineering, Faculty of Electronic and Information Engineering, Xi’an Jiaotong University, Xi’an, Shaanxi, China

**Keywords:** apple fruitlet, computer vision in agriculture, image dataset, natural scenes, target detection

## Abstract

This study introduces a small apple pre-thinning dataset designed to support the development of intelligent thinning systems by providing reliable data for small apple detection. The dataset comprises 2,517 RGB images (original size 3024×3024 pixels, uniformly resized to 500×500 pixels for standardization) systematically captured under real-world orchard conditions. The dataset encompasses natural variations in weather conditions (sunny/cloudy), lighting scenarios (direct sunlight/backlight), and fruit sizes (3-25mm diameter range) to ensure broad applicability. Each image was meticulously annotated using LabelImg software, with all small apple targets precisely labeled using both PASCAL VOC (XML) and YOLO (TXT) format bounding boxes, facilitating compatibility with various detection frameworks. Validation experiments conducted across multiple detection architectures (including Faster R-CNN, Cascade R-CNN, YOLO series, RT-DETR, DEIMv2, etc.) demonstrate the dataset’s effectiveness. This dataset serves as a valuable resource for developing intelligent thinning systems, with potential applications in promoting automation in the apple industry, enhancing thinning efficiency, and improving fruit quality.

## 1 Introduction

Fruit thinning constitutes an essential practice in modern precision orchard management, with direct implications for fruit quality, yield optimization, and economic profitability. In apple production, this operation is typically conducted 20–40 days after full bloom, corresponding to a developmental stage where young apples measure between 5–30 mm in diameter. The selective removal of fruitlets during this period is crucial for alleviating nutrient competition and promoting the optimal growth of remaining fruits (Ali, 2025). Nonetheless, conventional manual thinning practices remain heavily reliant on subjective empirical judgment, leading to inherent limitations including low efficiency, high labor costs, and inconsistent outcomes characterized by either missed or over-thinning (Xin et al., 2025). Against the backdrop of a rapidly worsening global agricultural labor shortage, the development of automated, vision-based thinning technologies has therefore emerged as an urgent priority. The cornerstone of such automated systems lies in the accurate and robust detection of small apples during the pre-thinning stage.

Deep learning-based approaches such as YOLO (Redmon et al., 2016) and DEIM (Huang et al., 2025a) have demonstrated remarkable success in various object detection applications. Their implementation has been extended to agricultural domains, including fruit detection (Xie et al., 2025; Jia et al., 2025), pest and weed identification (Suzauddola et al., 2025; Santhanambika and Maheswari, 2025; Betitame et al., 2025; Goyal et al., 2025), and automated harvesting (Jin et al., 2025). Nevertheless, the specific challenge of detecting small apples during the pre-thinning stage remains relatively under-explored. Since deep learning-based detection models depend on large-scale datasets for training, the absence of a dedicated, publicly available dataset for this particular task has significantly impeded research advancement. To address this gap, this study introduces a comprehensive dataset specifically designed for detecting small apples prior to thinning. The main contributions of this work are as follows:

1. Present a publicly available dataset for pre-thinning small apple detection. The dataset captures a wide range of real-world challenges, including scale variation, occlusion, and diverse lighting conditions.
2. Establish a rigorous data collection and annotation protocol, which incorporates a multi-stage quality control process to ensure high-quality annotations.
3. Provide extensive baseline evaluations by testing a suite of object detection models using standard COCO metrics, offering a critical reference for future research.

The paper is organized as follows: Section 1, Introduction, outlines the significance and contributions of this study. Section 2, Related work, reviews existing research and its limitations while highlighting the focus of our work. Section 3 elaborates on the value and key characteristics of the proposed dataset. Section 4 details the materials and methods, including the data acquisition setup, annotation process, benchmarking strategy, and experimental results. Section 5 acknowledges limitations and outlines directions for future research. Finally, Section 6 concludes the paper.

## 2 Related work

### 2.1 Deep learning-based object detection in agriculture​​

Deep learning has revolutionized visual perception in agriculture. Early applications primarily involved the direct adoption of generic object detection frameworks like Faster R-CNN (Ren et al., 2016) and SSD (Liu et al., 2016) for agricultural targets. However, these models often exhibited limited robustness when confronted with the inherent challenges of agricultural environments, including complex backgrounds, significant scale variation, and varying lighting conditions. To address these issues, subsequent research has focused on domain-specific architectural improvements. A prominent direction is the enhancement of feature pyramid networks (FPN) to better handle the multi-scale nature of agricultural objects, from small flowers to large fruits (Jia et al., 2021; Wang et al., 2025). Furthermore, the integration of attention mechanisms, such as convolutional block attention modules (CBAM) (Woo et al., 2018) and squeeze-and-excitation (SE) blocks (Hu et al., 2018), has been widely explored to improve feature representation in the presence of occlusion and clutter. More recently, Transformer-based architectures (Lou et al., 2025; Chen et al., 2025) have been introduced for their superior global context modeling capabilities, showing promising results in fruit detection (Guo et al., 2024) and counting tasks (Yang et al., 2023). Despite these algorithmic advances, the performance of deep learning models remains heavily dependent on the availability of large-scale, high-quality, and task-specific datasets.

### 2.2 Agricultural datasets for object detection​​

The availability of public datasets has been a key driver of progress in agricultural computer vision. Several benchmark datasets have been established for mature fruit detection, which is critical for harvesting robotics. Notable examples include the MinneApple dataset (Häni et al., 2020) for apple detection and the Deep Fruits dataset (Bargoti and Underwood, 2017). These datasets have facilitated the development and benchmarking of numerous detection algorithms. However, they are predominantly composed of images of mature or near-mature fruits, which are larger, exhibit more distinct color contrast against the foliage, and are often less densely clustered compared to fruits in the early growth stages. Consequently, models trained on these datasets are not directly applicable to the task of detecting small, green, and occluded fruitlets during the pre-thinning stage. While some research have begun to address earlier phenological stages, they often lack the scale, diversity of challenges, or public accessibility required for robust model development. This creates a significant data gap, specifically for the critical agricultural practice of fruit thinning.

### 2.3 The gap in pre-thinning small apple detection​​

The task of small apple detection prior to thinning presents unique challenges that are not adequately addressed by existing datasets. As summarized in **Table 1**, the target characteristics differ substantially from those of mature fruits. Pre-thinning small apples are typically defined by their small size, minimal color differentiation from the background foliage, and occurrence in dense clusters with mutual occlusion. These factors result in a domain shift that limits the applicability of models trained on mature fruit data. Although techniques like data augmentation and transfer learning can provide some improvements, they are insufficient to overcome the fundamental data distribution mismatch. Therefore, there is a pressing need for a dedicated, large-scale dataset that accurately captures the visual characteristics and challenges associated with the pre-thinning period. Such a dataset is essential for driving algorithmic innovation, enabling fair benchmarking, and ultimately facilitating the development of reliable vision systems for automated thinning.

**Table 1:** Comparison of key characteristics between mature fruit detection and pre-thinning small apple detection.

Characteristic	Mature fruit detection	Pre-thinning small apple detection
Primary application	Automated harvesting/Yield estimation	Automated thinning
Target size	Large (Relative to image)	Small (Area often < 32^2^ pixels)
Color contrast	High (Red to Green)	Low (Green-on-Green)
Representative datasets	MinneApple (Häni et al., 2020) Deep Fruits dataset (Bargoti and Underwood, 2017)	Small apple dataset (This work)

This work bridges this gap by introducing the small apple dataset, specifically designed to address the challenges of small apple detection during the thinning season. Our dataset not only provides the necessary data foundation but also establishes rigorous criteria to propel future research in this critical area.

## 3 Value of the data

### 3.1 Diverse orchard imagery

The dataset was manually captured in an experimental orchard. Images were acquired under varying natural lighting and weather conditions prior to fruit thinning, ensuring robust applicability for real-world agricultural scenarios. The diversity of the dataset improves the generalization capability of deep learning models in real orchard environments.

### 3.2 Ready-to-use annotations

All images were annotated using LabelImg and are provided in a format compatible with mainstream machine learning frameworks (e.g., PyTorch). This minimizes preprocessing efforts and accelerates deployment for AI-driven agricultural research.

### 3.3 Foundation for smart orchard research

This dataset serves as a resource for advancing computer vision applications in precision agriculture. It supports the development of machine learning models for apple fruitlet detection and early yield prediction, enabling data-driven orchard management.

### 3.4 Challenging small-target detection dataset in color-near scenes

This dataset presents a particularly valuable resource for investigating multi-scale object detection challenges, with special emphasis on small-target recognition in complex color-near environments. The dataset captures the unique challenge where apple fruitlets exhibit similar coloration to background foliage, making it ideal for: (i) developing robust detection algorithms for small, low-contrast targets in close-range agricultural scenes, and (ii) advancing research on occlusion handling in dense foliage environments. Beyond its primary application in apple fruitlet detection, the dataset serves as: (i) a resource for early-stage detection of various fruit species with similar color blending characteristics, and (ii) a critical resource for developing automated fruit thinning systems that must operate in visually complex orchard conditions.

## 4 Materials and methods

**Figure 1:**
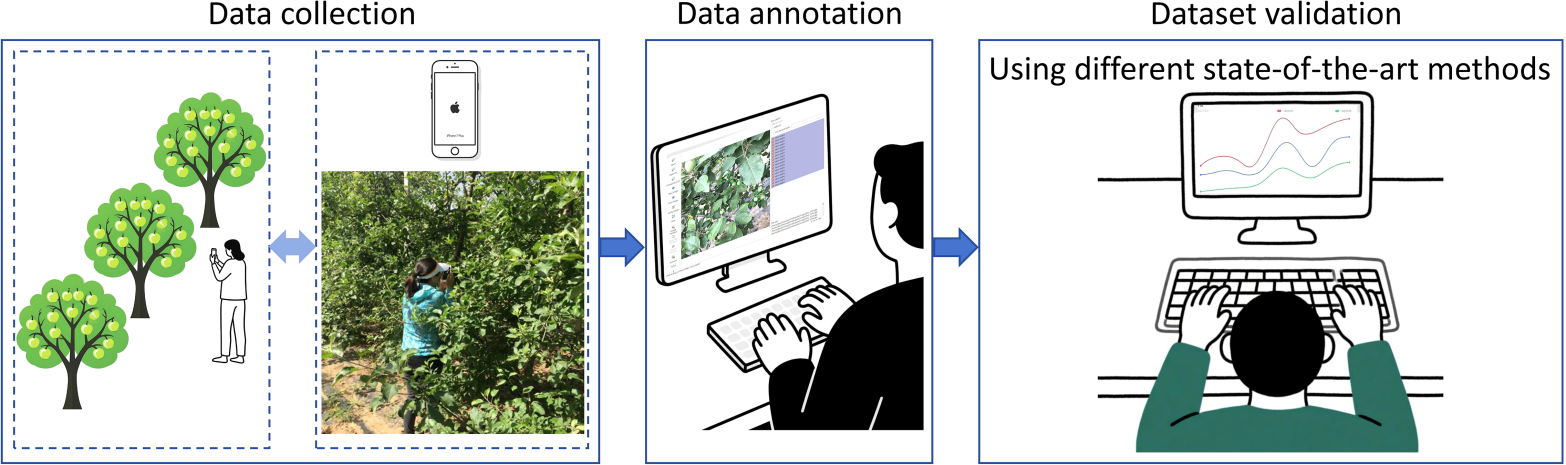
The flowchart of dataset construction.

The primary objective of this study is to construct a dataset for small apple detection prior to fruit thinning. The dataset development pipeline, illustrated in **Figure 1**, comprises three main stages: data collection, data annotation, and dataset validation. These stages will be detailed in the subsections below.

### 4.1 Data collection

The image dataset was collected from an experimental orchard at the College of Horticulture, Northwest A&F University (Yangling, Shaanxi, China). The dataset was captured using two mobile devices: an iPhone 7 Plus, equipped with a 12-megapixel CMOS sensor (f/1.8 aperture, hybrid autofocus, optical image stabilization), and an iPhone 6, equipped with an 8-megapixel CMOS sensor (f/2.2 aperture, hybrid autofocus). The comprehensive camera specifications are provided in **Table 2**. The data collection was designed to encapsulate a wide range of real-world orchard conditions, thereby enhancing the dataset’s robustness. Image acquisition was conducted during three distinct sessions in May 2018 under varying weather conditions: May 1 (sunny), May 2 (sunny), and May 4 (cloudy), with daily collection periods spanning 09:00-11:30 and 14:30-18:30 to capture diverse lighting conditions (backlight and direct sunlight). Complete data collection parameters are detailed in **Table 3**.

**Table 2:** Description of camera device.

Manufacture	Apple
Equipment	iPhone 7 Plus/ iPhone 6
Lens	28 mm
Sensor size	1/3 inch
Aperture	f/1.8 (iPhone 7 Plus)/ f/2.2 (iPhone 6)
Camera flash	No
Images	Size: 3024×3024 pixels (iPhone 7 Plus)/2448×2448 pixels (iPhone 6)
	Number: 1606 (iPhone 7 Plus)/ 911 (iPhone 6)
	Resolution: 72 dpi
	Bit depth: 24

**Table 3:** Description of data collection.

Fruit	Apple
Sample taking time	9:00-11:30 a.m. and 2:30-6:30 p.m. on May 1, May 2and May 4 in 2018
Location	Latitude: 34, Longitude:108
Climate	Sunny; Cloudy
Temperature	28-32°C approximately

A systematic protocol was followed to ensure comprehensive coverage and minimize bias:

#### 4.1.1 Plot selection

Data were collected from multiple rows of Fuji apple trees.

#### 4.1.2 Viewpoint and distance

The camera was positioned to simulate the viewpoint of an automated thinning robot, typically at a distance of 0.5 to 3 meters from the target canopy. The target trees had an approximate height range of 2.0-3.0 meters. A variety of angles, including horizontal, elevated, and top-down views, were employed to capture the fruit clusters from different perspectives.

#### 4.1.3 Scale variation

To account for the rapid growth of fruitlets, close-up shots of individual or small clusters of apples were taken alongside wider shots capturing the context within the canopy.

#### 4.1.4 Occlusion and complexity

Specific attention was paid to capturing images with varying degrees of occlusion, from fully visible apples to those heavily obscured by leaves, branches, or other fruits.

**Figure 2:**
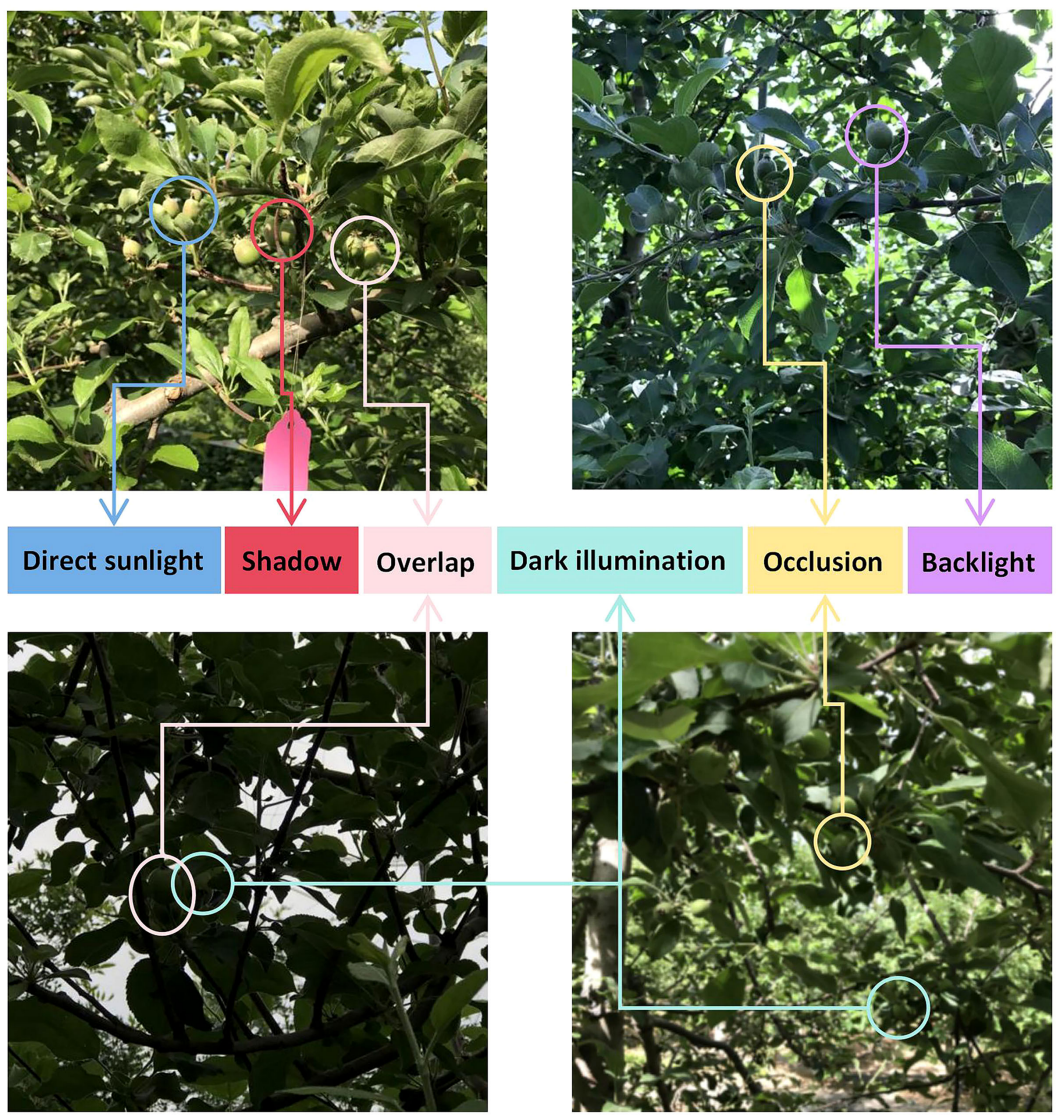
Representative examples of the captured images.

The image dataset was carefully designed to capture diverse field conditions, with samples acquired under varying natural daylight scenarios including both backlight and direct sunlight illumination. All images were stored in standard JPEG format with full preservation of the original 12-megapixel resolution. During acquisition, the apple fruitlets were in their early developmental stage, as evidenced by horizontal diameters measuring less than 25 mm. Representative examples of the captured images, demonstrating the range of lighting conditions and fruitlet characteristics, are presented in **Figure 2**.

In total, over 3,000 raw images were captured. Following an initial quality check to remove blurry or severely over/under-exposed images, a final set of 2,517 high-quality images was selected for annotation.

### 4.2 Data annotation

#### 4.2.1 Data annotation methodology

**Figure 3:**
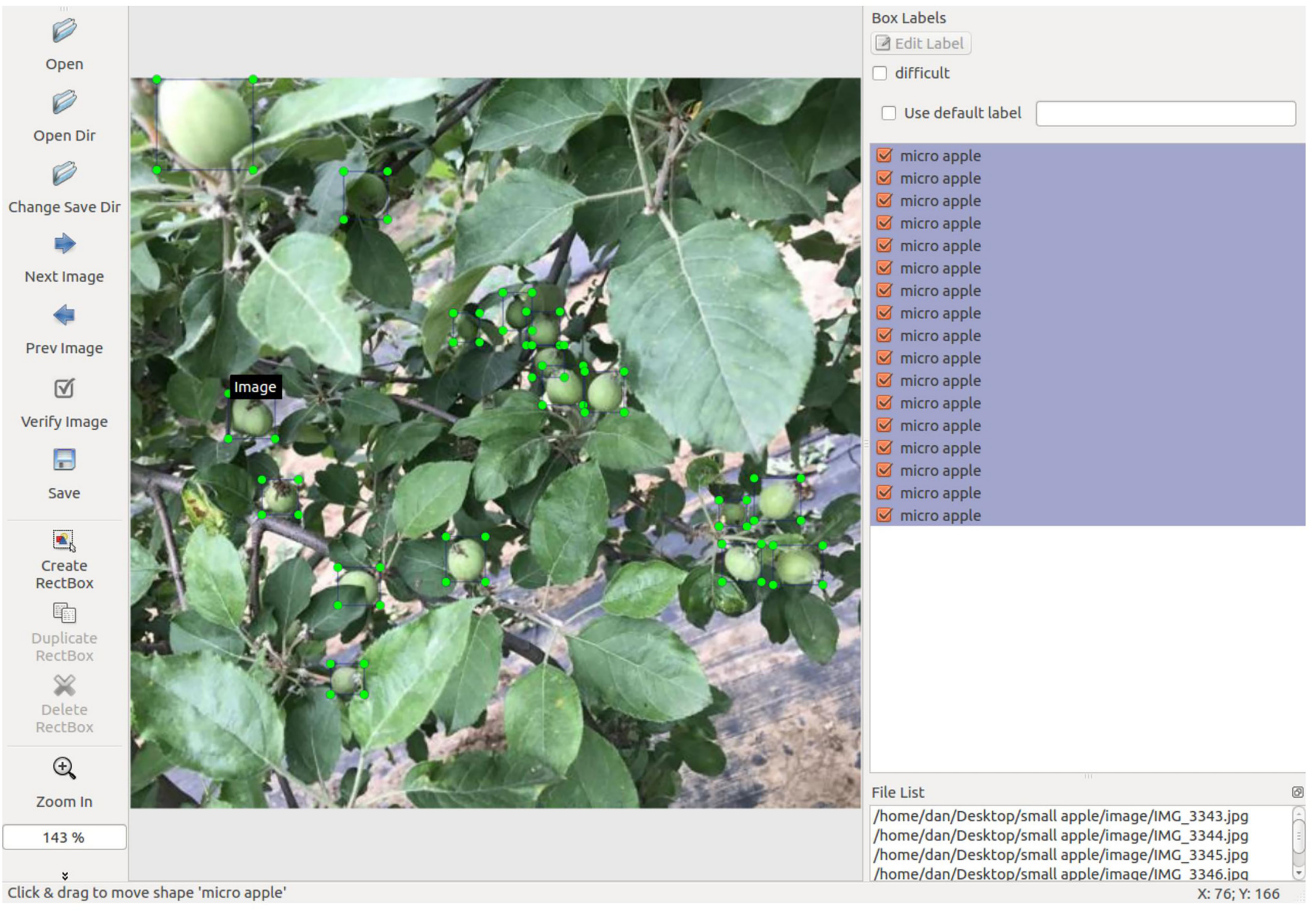
Representative annotation example.

All images in the dataset were uniformly rescaled to 500×500 pixels to ensure processing efficiency. Manual annotation was performed using LabelImg software, with bounding box coordinates stored in XML format. To enhance usability across different platforms, we additionally provide annotations in TXT format. **Figure 3** illustrates a representative annotation example. During the annotation process, special attention was given to: (1) precise localization of small fruitlets, and (2) accurate annotation of partially occluded targets, where only visible portions were labeled to minimize false positives in subsequent analyses.

#### 4.2.2 Quality control pipeline

In this study, a single annotator was responsible for the initial annotation to ensure consistency in style. To ensure dataset reliability, we implemented a rigorous quality control protocol following annotation completion. This involved comprehensive review of all labeled images and corresponding annotations to identify and rectify any missing or erroneous labels. Our quality control protocol consisted of three key phases:

##### 4.2.2.1 Phase 1

Iterative self-validation by the annotator. The annotation process was conducted in multiple batches. After completing each batch, the annotator would take a mandatory 24-hour break before re-reviewing 100% of the images in that batch. This “cooling-off” period was critical for allowing the annotator to approach the data with a fresh perspective, making it easier to spot initial oversights or inconsistencies. During this self-review, the annotator carefully verifying that every visible small apple was captured and that the bounding boxes were tightly fitted.

##### 4.2.2.2 Phase 2

Automated consistency checking. Following the self-validation, we ran a custom Python script to analyze the generated XML annotation files. This script checked for common errors that are difficult to catch manually across a large dataset. Checked items included:

1. Extremely small boxes: Flagging bounding boxes with a width or height of less than 5 pixels for manual re-inspection, as these could be annotation noise.
2. Invalid coordinates: Ensuring all bounding box coordinates were within the image boundaries.
3. Class label verification: Confirming that only the correct class label was present.

##### 4.2.2.3 Phase 3

Final expert adjudication and check. This was the most critical QC step. A senior agricultural expert independently reviewed 100% of the annotated images. The expert had the authority to correct any errors directly in the annotation files. This adjudicated version constitutes the final, released dataset. The error correction rate during this phase was found to be below 2%, indicating the high initial quality achieved by the previous phases.

#### 4.2.3 Dataset statistical characterization

A comprehensive statistical analysis was conducted to quantitatively characterize the composition and key challenges present in the proposed dataset. This analysis aims to provide a transparent overview of the data, facilitating a deeper understanding of its properties and the difficulties it presents for detection models. The statistical characterization of the dataset are shown in **Table 4** and the details are as follows.

**Table 4:** Statistical characterization of the dataset.

Conditions	Sub-category	Number of images	Percentage	Number of targets	Percentage
Weather	Sunny	1,670	66.3%	13,089	58.4%
	Cloudy	847	33.7%	9,326	41.6%
Lighting	Direct sunlight	1,181	46.9%	10,656	47.5%
	Backlight	1,336	53.1%	11,759	52.5%
Acquisition time	09:00-11:30	996	39.6%	9,983	44.5%
	14:30-18:30	1,521	60.4%	12,432	55.5%
Target size distribution (Area of annotated bounding box)	Area < 32² pixels	2,108	83.8%	14,080	62.8%
	32² pixels ≤ Area < 96² pixels	2,069	82.2%	8,230	36.7%
	Area ≥ 96² pixels	68	0.3%	105	0.5%

##### 4.2.3.1 Target statistics

The dataset comprises a total of 2,517 RGB images. A cumulative of 22,415 small apple fruitlets with different conditions were meticulously annotated.

##### 4.2.3.2 Environmental condition distribution

The dataset was constructed to encompass a diverse range of real-world conditions. The distribution of images across different weather and lighting scenarios is summarized in **Table 4**. This deliberate variation ensures that models trained on the dataset are exposed to a wide spectrum of visual appearances, thereby enhancing their potential robustness.

##### 4.2.3.3 Temporal distribution

The distribution of images across the time of day (morning and afternoon) is shown in **Table 4** to further characterize the data composition.

##### 4.2.3.4 Object scale distribution

The scale of objects is a critical factor, especially for small object detection. The distribution of bounding box areas (in pixels) for all annotated targets is illustrated in **Table 4**. Notably, over 60% of the bounding boxes have an area smaller than 32^2^ pixels, formally categorizing them as small objects according to the COCO benchmark criteria. This distribution confirms the dataset’s relevance to the core challenge of small object detection.

In summary, the statistical characterization confirms that the dataset not only provides a substantial number of targets but also encapsulates the primary challenges of small apple detection: small object size, and environmental variation. These quantified attributes are significant for evaluating the robustness of object detection algorithms in real-world orchard settings.

### 4.3 Dataset validation

The dataset supports comprehensive preprocessing and partitioning for machine learning applications. Researchers may perform data augmentation through various image transformations, including noise injection, brightness adjustment, chromaticity modification, contrast variation, and sharpness alteration. For model development and evaluation, the dataset can be partitioned into training, validation, and test subsets using multiple ratio configurations (8:1:1, 7:2:1, or 6:2:2), providing flexibility for different experimental designs and ensuring robust evaluation of deep learning models.

#### 4.3.1 Dataset validation methods​

To rigorously evaluate dataset effectiveness, we conducted evaluations using ten representative object detection architectures spanning different paradigms: (1) three two-stage detectors (Faster R-CNN (Ren et al., 2016), Cascade R-CNN (Cai and Vasconcelos, 2018), and Grid R-CNN (Lu et al., 2019)) and (2) seven single-stage detectors (RetinaNet (Lin et al., 2017), YOLOv5 (Jocher et al., 2020), YOLOv8, YOLOv11 (Khanam and Hussain, 2024), YOLOv12 (Tian et al., 2025), RT-DETR (Zhao et al., 2024), and DEIMv2 (Huang et al., 2025b)). This multi-model validation approach ensures robust assessment of the dataset’s suitability for various detection paradigms.

The dataset was randomly partitioned into training (2,013 images), validation (253 images), and test (251 images) sets at an 8:1:1 ratio for model training. The random partitioning strategy was intentionally employed to demonstrate dataset generalizability and support reliable performance evaluation across different experimental configurations.

#### 4.3.2 Hardware and software platform

All experiments were conducted on a workstation equipped with an Intel Core i9-11900H CPU, 32GB of RAM, and an NVIDIA GeForce RTX 3080 GPU (16GB VRAM), running on a Windows 10 operating system. The software environment consisted of Python 3.8, PyTorch 2.2.2, and CUDA 11.8 for GPU acceleration.

#### 4.3.3 Performance of different methods

All experimental results are meticulously documented and presented in **Table 5**. This table provides a comprehensive evaluation of ten object detection models on our apple fruitlet dataset, assessed using standard COCO metrics: Average Precision (AP) and Average Recall (AR). The experimental results reveal significant performance disparities among the models. The Transformer-based RT-DETR-L model leads in overall accuracy (AP = 0.669), demonstrating the most robust overall detection capabilities. In contrast, DEIMv2-N excels in recall (AR = 0.706) and loose-threshold precision (AP@0.5 = 0.921), offering particular value for applications like fruit thinning where a high recall rate is critical. Modern YOLO series models (v8, v11, v12) provide a balanced and high-performance alternative. Notably, the accuracy for small targets (AP_S) is substantially lower than for medium and large targets across all models. This performance gap further validates the inherent difficulty of detecting small apples prior to thinning and underscores the challenging nature of the presented dataset. Collectively, the benchmarking results from these ten models conclusively validate the effectiveness and utility of the proposed dataset for the challenging task of small apple detection.

**Table 5:** Results of different methods on detecting apple fruitlets.

Methods	AP	AP@0.5	AP@0.75	AP_S	AP_M	AP_L	AR	AR_S	AR_M	AR_L
Faster R-CNN (ResNet50)	0.492	0.773	0.569	0.401	0.647	0.611	0.575	0.500	0.711	0.637
Cascade R-CNN (ResNet50)	0.523	0.784	0.608	0.416	0.694	0.701	0.604	0.524	0.747	0.731
Grid R-CNN (ResNet50)	0.566	0.828	0.670	0.465	0.725	0.832	0.641	0.564	0.776	0.869
RetinaNet	0.441	0.709	0.489	0.344	0.605	0.481	0.549	0.465	0.700	0.519
YOLOv5-N	0.619	0.833	0.743	0.506	0.794	0.880	0.652	0.552	0.826	0.912
YOLOv8-N	0.635	0.853	0.761	0.525	0.799	0.896	0.668	0.575	0.831	0.925
YOLOv11-N	0.638	0.852	0.762	0.532	0.799	0.921	0.670	0.578	0.829	0.931
YOLOv12-N	0.634	0.854	0.752	0.529	0.797	0.907	0.666	0.572	0.829	0.938
RT-DETR-L	0.669	0.890	0.800	0.577	0.806	0.935	0.702	0.626	0.834	0.956
DEIMv2-N	0.652	0.921	0.764	0.550	0.797	0.943	0.706	0.634	0.830	0.963

Beyond its immediate application to CNN-based models, the dataset holds significant potential for advancing state-of-the-art methodologies. Firstly, the high-quality manual annotations of the dataset make it particularly suitable for training and evaluating modern approaches such as self-supervised and unsupervised learning models, which require substantial amounts of data to learn meaningful representations without exhaustive manual labels. Secondly, the complexity and variety of scenes challenge unsupervised object detection algorithms, pushing the boundaries of models that can identify and segment objects without prior knowledge.

## 5 Limitations

The primary limitation of this dataset is its origin from a single experimental station, which may limit the generalizability of models trained on it to commercial orchards operating under different geographical and managerial conditions. However, the dataset was explicitly designed to capture a wide spectrum of visual challenges (e.g., lighting variations, occlusion levels, scale changes) inherent to the task. To address the current geographical limitation, our immediate next step is to enrich the dataset with samples from a wider range of regions and cultivation systems. This expansion will, in turn, fuel our investigation into domain adaptation methods designed to ensure model performance and reliability in unfamiliar orchard environments.

Additional limitations include the exclusive composition of the dataset with Fuji apple instances, which may hinder the model’s generalization to other apple varieties or fruit species with significantly different morphological characteristics. Future work should therefore incorporate more diverse cultivars and species to enhance the robustness and broader applicability of the approach.

## Data Availability

The datasets presented in this study can be found in online repositories https://dx.doi.org/10.21227/z74d-8t41.
